# From Where We've Come to Where We Need to Go: Physiotherapy Management of Chronic Whiplash-Associated Disorder

**DOI:** 10.3389/fpain.2021.795369

**Published:** 2022-01-07

**Authors:** Cameron Dickson, Rutger M. J. de Zoete, Tasha R. Stanton

**Affiliations:** ^1^School of Allied Health Science and Practice, The University of Adelaide, Adelaide, SA, Australia; ^2^Innovation, Implementation and Clinical Translation in Health, University of South Australia, Adelaide, SA, Australia

**Keywords:** whiplash-associated disorder (WAD), neck pain, chronic pain, physiotherapy, evidence-based practice (EBP)

## Introduction

Chronic neck and back pain are the leading causes of years-lived-with-disability globally (1). Neck pain due to traumatic onset is commonly classified as whiplash-associated disorder (WAD), and is associated with more severe symptom presentations (2) than non-traumatic neck pain. Recovery rates for WAD are around 50%, with ~30% of patients developing severe disability (3). Individuals with chronic WAD present with altered psychological status, widespread sensory hypersensitivity, and motor system dysfunction (4–6). Here we reflect on advances in our understanding and management of WAD and generate discussion around ongoing systemic challenges faced by physiotherapists managing these patients.

## Physiotherapeutic Management of WAD

Large strides have been made in physiotherapy management of WAD. Our treatments are more evidence-based. We have moved from regular use of electrotherapeutic modalities to active management, which is now more thoroughly integrated into patient care (7). Exercise (8), cognitive functional therapy (9), and pain science education (10) are commonplace, and heightened through the delivery of psychologically-informed physiotherapy (11). Physiotherapy is now more multi-disciplinary and “bio-psycho-social,” having recognized the importance of such an approach for WAD, which has been traditionally considered a purely physical complaint.

Prescription and monitoring of our treatments have also improved. For example, moderate non-adherence to home-based exercise programs (1, 12) negatively influences patient outcomes (13) in people with pain. Technological advancements, such as the use of smartphone technology, have helped overcome barriers to adherence, with evidence supporting their use for behavior change (14). Exercise programs can now be provided via ‘Apps,' including the ability to electronically track compliance and progress. Patients can also view video clips of exercises and log their adherence and outcomes, enhancing engagement and care (15). Additionally, extension to internet-based physiotherapy interventions for WAD is beginning to grow, with an internet-delivered behavioral programme showing comparative efficacy as face-to-face intervention (16).

The importance of communication in the management of WAD is also now better understood (10). As demonstrated in low back pain research, typical expressions such as “wear and tear” and “disc space loss” are often interpreted by patients to mean their spine is “crumbling” or “collapsing” [(17), p. E1120]. This has underpinned more careful and deliberate approaches to communication, given that such beliefs inform perceptions of a poor prognosis, which has been linked to poorer recovery (18, 19). We also now understand that over-diagnosis (i.e., “the detection of abnormalities that are not destined to ever bother us” [(20), p. 27]) can be iatrogenic (21) particularly given our increased understanding that there are various contributors to pain, of which perceived damage informed by imaging may well-contribute. This is reflected in widespread use of the cognitive functional model which posits an integrative approach considering pathoanatomical, physical, cognitive, emotional, social, health, sensory and lifestyle factors (9). Given these relevant findings from spinal pain, consideration of these issues, such as potential iatrogenic harm related to imaging, within the context of WAD appears warranted. Regardless, the necessity for physiotherapists to possess new skills in the discussion of imaging findings, and exploration of individually specific factors contributing to WAD, is evident. Underpinning this is the ability to create a strong therapeutic alliance, utilize advanced communication skills, and hold an understanding of both neuroscience and behavioral psychology (9).

## Current Systemic Challenges in Managing WAD

Despite advances in our understanding of how to clinically approach WAD, systemic challenges impeding the implementation of best practice care remain. Increasingly, private health care is becoming consumer-driven. Whilst a patient-centered approach has evidence for improved WAD treatment, such an approach can be influenced by the patient's treatment expectations. In a large cohort of people with low back pain, 90% had expectations of service (e.g., specific diagnosis, investigations) contradictory to international clinical practice guidelines (22), and is likely a consideration for patients with WAD also. Furthermore, qualitative work highlights that people with acute WAD hold a biomechanical understanding of their condition, and believe that an X-ray is important for diagnosis; again, in contrast with best practice guidelines (23). We have a responsibility to educate our patients on best practice care for WAD and help them achieve their optimal recovery. However, this is not always an easy sell. Indeed, people with WAD report frustration with treatment focus on psychological aspects of WAD, instead valuing clear information about physical diagnosis and prognosis (23).

The ability for physiotherapists to help patients reconceptualize their WAD as having numerous contributors, rather than biomechanical and structural contributors only, is also hampered by persistently high rates of inappropriate imaging. Numerous studies demonstrate that regularly reported ‘abnormal' features on magnetic resonance imaging (MRI), such as degenerative spine disease, facet joint arthropathy and disc protrusion, are common in people who are asymptomatic (24–26). Thus, potential exists for reported findings (which may largely be irrelevant to a patient's clinical presentation), to evoke negative beliefs in the patient about their injury or prognosis (19). Such beliefs can give rise to poorer clinical outcomes, and add to the challenge of progressing patients with persistent pain (27, 28). Despite the importance of thoughtful and sparing referral for imaging being apparent for several years, clinical practice has seemingly not changed (29–31). Imaging remains over-utilized in patients with WAD, with a significant proportion of scans ordered by general practitioners for WAD being inappropriate (29, 32). Indeed, a cross-sectional survey of Australian General Practitioners (*n* = 423) highlighted knowledge gaps in key criteria for appropriate imaging referral for WAD (33). For physiotherapists, systemic issues related to over-imaging add to the already difficult task of fostering helpful pain-related attitudes and beliefs amongst patients. That said, contemporary literature does reflect an interest in fatty infiltration of deep cervical extensor musculature amongst persons with WAD, as diagnosed via MRI and ultrasound (34, 35), and inflammation of the cervical spine detectable by advanced combined positron emission/computed tomography (36). Such research and implementation of imaging may hold promise in terms of identifying potential structural contributors to persistent pain and treatment targets, which may also serve patients well in terms of validating their symptoms. However, the clinical utility (and influence on physiotherapy practice) of such imaging findings remains unclear at this time. At present, we posit that the priority for imaging remains attempting to reduce non-guideline concordant imaging for WAD, given the known propensity for iatrogenic outcomes and increased costs (37).

Evidence supports the notion that pain is an “emergent” phenomenon, reflecting perception of threat, as opposed to solely reflecting tissue damage (38). However, reconceptualizing pain in this way presents a considerable challenge. The competence of clinicians to work through psychosocial elements with patients, the necessity to move away from typical passive interventions, and time constraints of consults have all been identified as barriers to the successful integration of this model (39). Pragmatically, issues around time constraints are not an easy fix. Many private physiotherapists work with 20 to 30-min consults, seeing several patients in a row. Yet, evidence supporting pain education for WAD typically involves sessions up to several hours in duration (10). Consult time pressure has also been identified as a potential barrier to the reduction of unnecessary medical care (e.g., referral for imaging); and seems a likely influence on physiotherapy practice. In the interest of providing high-quality evidence-based care for patients with WAD do we double the consult duration, and double the fee? How does this sit with the price-sensitive consumer? Some commercial challenges can be foreseen in terms of getting a meaningful ‘buy-in' from private sector patients such that outcomes can be enhanced. This issue also extends to private health insurance providers and the compensable injury sector, in terms of their preparedness to invest in such an approach.

Commission-based pay structures for private physiotherapists may also play into persistent pain. Self-management strategies and active rehabilitation are fundamental to treating WAD, as opposed to more traditional and passive ‘in-rooms' modalities, which is arguably in contrast to a privately employed clinician's financial interests. Fostering co-dependence consciously or otherwise (i.e., through overtreatment or overutilization) has the potential to adversely impact a patient's clinical outcome, and perhaps presents a systemic hurdle to implementing best practice (21). Does consideration need to be given to alternate reimbursement structures for physiotherapists? Formal exploration of this point by stakeholders and governing bodies seems a worthwhile initiative.

## Moving Forward

Most physiotherapists would be no stranger to a patient's perception that if hands-on manual therapy was not provided, then they have not been ‘treated.' Yet such an approach is frequently not optimal care for someone with WAD. Reducing pre-conceived ideas in the community of what physiotherapy comprises is essential to improve patient engagement, create greater scope for the integration of best practice care, and optimize the prospects of patients achieving their health goals.

The expectations of patients regarding their recovery from WAD are also vitally important in terms of prognosis, highlighting the need to provide patients with quality, evidence-informed information (40). Albeit within a simulated-patient cohort with low back pain, this point is supported by recent work demonstrating that the perceptions of patients with recent onset spinal pain were more positive when provided with best practice advice (i.e., advice to keep moving as tolerated, no imaging, and a positive prognosis), than when imaging was provided (41). And in people who were provided imaging reports, perceptions were more positive when provided with advanced reporting—inclusive of typical age-specific imaging findings (41). Similarly, advanced reporting has also been linked to reduced prescription of narcotics for back pain (42).

While excellent work continues to explore better implementation of ‘high-value' care (i.e., care that is consistent with evidence-based guidelines) for physiotherapy management of WAD (3, 7, 21), if we are to continue to advance our care, and the outcomes of our patients, some considerable systemic challenges must be overcome. There appears a critical need for greater synergy between General Practitioners, physiotherapists, medical imaging providers, specialists, and other allied health professions such that we could approach the societal burden of WAD in partnership, rather than as a fragmented system (43). At ground level, this could look like a more formalized alliance between the professions—from which closer relations, common professional development, shared input and debate, and consistency of messaging for patients could result. This could provide a platform for more robust, evidence-based clinical care, and reduce any inadvertent iatrogenic influences on chronic pain development. The importance of formalized alliances is highlighted by a recent systematic review pertaining to the health-related information needs of persons with chronic spinal pain (44). Two predominant themes were identified: a desire for a ‘definitive diagnosis' and potential imaging, and ‘clear, trustworthy, consistent information' (44). Herein lies both the challenge and perhaps the answer. To counter such unhelpful beliefs around structural diagnosis and imaging, it is essential to have all care providers delivering robust, evidence-based, and *consistent* messages.

## Conclusion

The standards and efficacy of physiotherapy practice have improved markedly over the years—which is something to be celebrated. However, systemic influences are an impediment to the optimal management of WAD. We argue that the public health challenge of WAD requires a multi-faceted public health response, as highlighted here.

## Author Contributions

All authors listed have made a substantial, direct, and intellectual contribution to the work and approved it for publication.

## Funding

TS is supported by a National Health & Medical Research Council of Australia Career Development Fellowship (ID1141735).

## Conflict of Interest

TS has received payment for lectures relating to pain and rehabilitation. The remaining authors declare that the research was conducted in the absence of any commercial or financial relationships that could be construed as a potential conflict of interest.

## Publisher's Note

All claims expressed in this article are solely those of the authors and do not necessarily represent those of their affiliated organizations, or those of the publisher, the editors and the reviewers. Any product that may be evaluated in this article, or claim that may be made by its manufacturer, is not guaranteed or endorsed by the publisher.
